# Moderators of the Effect of a Self-directed Digitally Delivered Exercise Program for People With Knee Osteoarthritis: Exploratory Analysis of a Randomized Controlled Trial

**DOI:** 10.2196/30768

**Published:** 2021-10-29

**Authors:** Rachel K Nelligan, Rana S Hinman, Fiona McManus, Karen E Lamb, Kim L Bennell

**Affiliations:** 1 Centre for Health, Exercise and Sports Medicine, Department of Physiotherapy School of Health Sciences The University of Melbourne Parkville Australia; 2 Centre for Epidemiology and Biostatistics Melbourne School of Population and Global Health The University of Melbourne Parkville Australia; 3 Methods and Implementation Support for Clinical Health research platform Faculty of Medicine, Dentistry and Health Sciences The University of Melbourne Parkville Australia

**Keywords:** digital, text messaging, exercise, moderators, osteoarthritis, RCT, clinical trial, subgroups, pain, function, knee osteoarthritis, rehabilitation, digital health

## Abstract

**Background:**

A 24-week self-directed digitally delivered intervention was found to improve pain and function in people with knee osteoarthritis (OA). However, it is possible that this intervention may be better suited to certain subgroups of people with knee OA compared to others.

**Objective:**

The aim of this study was to explore whether certain individual baseline characteristics moderate the effects of a self-directed digitally delivered intervention on changes in pain and function over 24 weeks in people with knee OA.

**Methods:**

An exploratory analysis was conducted on data from a randomized controlled trial involving 206 people with a clinical diagnosis of knee OA. This trial compared a self-directed digitally delivered intervention comprising of web-based education, exercise, and physical activity program supported by automated exercise behavior change mobile phone text messages to web-based education alone (control). The primary outcomes were changes in overall knee pain (assessed on an 11-point numerical rating scale) and physical function (assessed using the Western Ontario and McMaster Universities Osteoarthritis Index function subscale [WOMAC]) at 24 weeks. Five baseline patient characteristics were selected as the potential moderators: (1) number of comorbidities, (2) number of other painful joints, (3) pain self-efficacy, (4) exercise self-efficacy, and (5) self-perceived importance of exercise. Separate linear regression models for each primary outcome and each potential moderator were fit, including treatment group, moderator, and interaction between treatment group and moderator, adjusting for the outcome at baseline.

**Results:**

There was evidence that pain self-efficacy moderated the effect of the intervention on physical function compared to the control at 24 weeks (interaction *P*=.02). Posthoc assessment of the mean change in WOMAC function by treatment arm showed that each 1-unit increase in baseline pain self-efficacy was associated with a 1.52 (95% CI 0.27 to 2.78) unit improvement in the control group. In contrast, a reduction of 0.62 (95% CI –1.93 to 0.68) units was observed in the intervention group with each unit increase in pain self-efficacy. There was only weak evidence that pain self-efficacy moderated the effect of the intervention on pain and that number of comorbidities, number of other painful joints, exercise self-efficacy, or exercise importance moderated the effect of the intervention on pain or function.

**Conclusions:**

With the exception of pain self-efficacy, which moderated changes in function but not pain, we found limited evidence that our selected baseline patient characteristics moderated intervention outcomes. This indicates that people with a range of baseline characteristics respond similarly to the unsupervised digitally delivered exercise intervention. As these findings are exploratory in nature, they require confirmation in future studies.

## Introduction

Osteoarthritis (OA) is a condition of the synovial joints [1], with the knee being commonly affected [2]. Knee OA causes joint pain and stiffness, which can often lead to reduced physical function and quality of life [3,4]. Knee OA is a highly prevalent condition and a leading contributor to disability globally [5]. Exercise is the core treatment in the management of knee OA, which is recommended in all clinical guidelines [6-9]. However, the effects of exercise on knee OA pain and function are modest overall [10]. This may be due to the existence of subgroups of people with certain baseline characteristics that cause them to respond in different ways to exercise [11]. Baseline patient characteristics that affect how a patient responds to treatment are called moderators [12]. A better understanding of potential moderators of the effects of exercise in knee OA will enable the identification of subgroups of people who respond more or less favorably to exercise treatments. This will facilitate the targeting of exercise treatments in knee OA and thus, may improve the effects of exercise on patient outcomes. The evaluation and identification of moderators of treatment effects has been named a major research priority in OA clinical guidelines [9,13].

The use of digitally delivered interventions to support chronic condition management is rapidly increasing as a means of improving access to evidence-based health care [14]. Recently, we developed and evaluated a 24-week self-directed digitally delivered intervention, designed to support people with knee OA to access and participate in an evidence-based exercise program [15]. In a randomized controlled trial (RCT), we found that this intervention led to greater improvements in pain and function compared to an education control at 24 weeks in people with knee OA. We also found that 72% and 68% of the participants in the intervention group (compared to 42% and 41% in the control group) experienced clinically meaningful improvements in pain and function, respectively. This demonstrates that most, but not all, people benefited from the intervention and could indicate the existence of subgroups of people who responded more (or less) favorably to the unsupervised, digitally delivered exercise intervention.

To our knowledge, no previous studies have conducted formal moderation analyses to explore baseline patient characteristics associated with the effect of self-directed digitally delivered exercise for people with OA although 2 studies have explored baseline characteristics as predictors of outcomes from self-directed exercise. One study identified that increased age and the presence of a comorbidity at baseline predicted nonusage of a self-directed web-based physical activity intervention for patients with knee and hip OA [16]. Another study found that the presence of comorbidity was associated with lower physical activity levels, while greater baseline arthritis self-efficacy was associated with greater physical activity, following a 12-week self-directed exercise program in adults with arthritis [17]. As these studies only examined associations between baseline characteristics and outcomes in the intervention arms with no control group comparisons, it is possible that these findings could have occurred, regardless of the interventions received. Therefore, these findings do not enable identification of potential subgroups of people who benefit (or not) from self-directed exercise interventions.

This exploratory study sought to identify moderators of the effect of a self-directed digitally delivered exercise intervention on changes in pain and physical function at 24 weeks relative to the control in people with knee OA. The findings of this study will address a key knee OA research priority and provide direction for future confirmatory studies.

## Methods

### Study Design

We conducted exploratory moderation analyses [18] by using data from a two-arm participant-blinded and assessor-blinded RCT [15]. The RCT evaluated the effects of self-directed digitally delivered exercise compared to an education control. Limited disclosure was used to blind the participants. All participants provided consent prior to enrolment into the RCT, which included the use of their deidentified data in secondary analysis. The RCT was approved by the University of Melbourne Human Research Ethics Committee (1851085) and prospectively registered (ACTRN12618001167257).

### Participants

In the RCT, 206 people with knee OA were recruited from the Australia-wide community via internet sources (social media and web-based newspapers) and a volunteer database. Full RCT eligibility criteria are reported elsewhere [19] and included ≥45 years of age, a clinical diagnosis of knee OA, and internet access.

### Intervention

Full details of the self-directed digitally delivered exercise intervention have previously been published [19,20]. To summarize, participants in the intervention received access to the same standardized custom-built website, “My Knee Exercise” and received a 24-week automated behavior change mobile phone text messaging program. The website was developed by the researchers (RKN, KLB, RSH) and feedback was provided by 3 people with knee OA. The website contained (1) educational information about OA, exercise, and sought to address common misconceptions about OA, (2) prescribed a 24-week lower limb strengthening exercise regimen, and (3) provided general physical activity guidance. The 24-week strengthening exercise regimen was divided into 3 programs, each of 8-weeks duration. The website advised that the 3 programs be completed consecutively. Each program contained 5-6 exercises. Participants were asked in the website to perform these exercises 3 times per week. The strengthening exercises focused on the hip, knee, and ankle (eg, sit-to-stand, seated knee extension, calf raise). Detailed exercise instructions, including when and how to increase an exercise challenge, were provided in both text and visual formats (photo, video) and were available to download. Exercise equipment (eg, ankle weights, resistance bands) was recommended, and suggestions about where to purchase equipment was provided. Exercise and physical activity logbooks were also provided and available to download.

Augmenting the strengthening exercise regimen was a 24-week automated exercise behavior change mobile phone text messaging program. The text messages were designed to monitor weekly exercise session completion and address exercise facilitators and barriers commonly encountered by people with knee OA. Program development systematically followed the Behavior Change Wheel Framework, which is a universally accepted approach to designing behavior change interventions [21]. The program functioned by prompting self-report of how many strengthening exercise sessions were completed in the previous week (each Monday initially, reducing to fortnightly by 24 weeks) and then provided tailored support depending on the level of exercise adherence (≥3 exercise sessions/week = adherent). Participants who self-reported ≥3 exercise sessions/week received a positive reinforcement message. Participants self-reporting <3 exercise sessions/week received a follow-up message asking them to select 1 reason (from a prespecified list of exercise barriers), which best explained the reason for <3 exercise sessions/week. Participants then received a message containing a behavior change suggestion linked to their selected barrier. Participants also received regular messages designed to facilitate ≥3 exercise sessions/week (twice weekly initially, reducing to fortnight by week 24). Dependent on weekly responses, participants received on average 2-5 messages per week. The frequency of messages sent to participants was designed to decline over the 24 weeks.

After randomization and enrolment into the study, intervention participants received an email containing website access, information about receiving text messages, the recommendation to access the website within a week to commence their exercise program, and were told they could continue to access the website at any time over the 24 weeks. Participants also received a text message encouraging website access. The control group received access to another custom-built website containing the same educational information as the intervention website. After randomization and enrolment into the study, control participants received an email containing website access, the recommendation to access the website within a week, and were told they could access the website at any time after the 24 weeks. Participants also received a single text message encouraging website access.

### Dependent Variables

All RCT outcomes were participant-reported and collected via REDCap electronic surveys at baseline and at 24 weeks. The 2 primary outcomes were (1) overall pain in the last week, measured using an 11-point numerical rating scale (terminal descriptors, 0=no pain to 10=worst pain possible) and (2) physical function, measured using the physical function subscale of the Western Ontario and McMaster Universities Osteoarthritis Index (WOMAC) (score range 0=no dysfunction, 68=maximum dysfunction). These measures are reliable and valid measures recommended for knee OA clinical trials [22-24]. At 24 weeks, the change in pain and function was calculated as baseline minus 24-week values of each.

### Selected Moderators

The selection of moderators was based on evidence and theoretical rationale and involved a review of the literature [16,17,25-29] and consensus by all authors (Multimedia Appendix 1). Five baseline variables were selected.

#### Number of Comorbidities

Number of comorbidities was collected via a question asking participants to select from a list of 13 comorbidities any of which were relevant to them (see Textbox 1). A participant’s selected comorbidities were then added to create a continuous score of the total number of comorbidities per participant. This resulted in a score range of 0 to 4.

**List of comorbidities.**

1. Heart disease (eg, angina, heart attack, heart failure)

2. High blood pressure

3. Problems caused by a stroke

4. Leg pain when walking due to poor circulation

5. Lung disease (eg, asthma, chronic bronchitis, emphysema)

6. Diabetes

7. Kidney disease

8. Diseases of the nervous system (eg, Parkinson disease, multiple sclerosis)

9. Liver disease

10. Cancer (within the last 5 years)

11. Depression

12. Arthritis in your back or other condition affecting your spine

13. Rheumatoid arthritis or another kind of arthritis in addition to osteoarthritis

#### Number of Other Joints With Pain

The number of other joints with pain was collected via a question asking participants to select any other joint they currently experience pain in, from a list of 9 joints, with responses converted into a continuous score of number of other joints with pain per participant. This resulted in a score range of 0 to 9.

#### Pain Self-efficacy

Pain self-efficacy relates to one’s confidence in their ability to control or manage pain [30,31]. Pain self-efficacy was measured using the pain subscale of the Arthritis Self-Efficacy Scale. The score range of the pain subscale is 1 to 10, with higher scores indicating greater pain self-efficacy [31].

#### Exercise Self-efficacy

Exercise self-efficacy relates to one’s ability to continue exercising in the face of barriers to exercise [32]. Exercise self-efficacy was measured using the Self-efficacy for Exercise Scale. The score range for this scale is 0 to 90, with higher scores indicating greater exercise self-efficacy [32].

#### Self-perceived Importance of Exercise

Exercise importance was measured in response to the question “How important is it to you to do regular exercise to manage your knee condition?” Responses were collected using a 7-point Likert scale with a score range of 1 to 7, with higher scores indicating greater importance.

### Statistical Analysis

Separate linear regression models were fit for each primary outcome with the baseline of the relevant outcome, treatment group, and one of the 5 potential moderators as covariates, including an interaction between treatment group and the relevant potential moderator. Results were calculated as the estimated mean effect of a 1-unit increase in the potential moderator for each treatment group. Using complete case data, fractional polynomials were employed to determine if nonlinear interaction models were warranted. All other analyses were performed on complete case data and multiply imputed data, including assessing regression assumptions of linearity and homoscedasticity using standard diagnostic plots. These potential moderator interactions were also assessed visually via plots of the difference in the change in the primary outcomes between groups versus the potential moderator. Multiply imputed data were the primary analysis in all interpretations. All statistical analyses were performed using Stata version 16.1 (StataCorp LLC, College Station).

## Results

### Baseline Descriptive Information

In this study, 206 people with a clinical diagnosis of knee OA were recruited from all Australian states and territories and were enrolled into the study (126/206, 61.2% female, mean age 60 [SD 8.4] years). Baseline characteristics of participants in both groups were similar (Table 1). At the 24-week follow-up, 88.3% (91/103) and 87.3% (90/103) of the participants in the intervention group and 89.3% (92/103) and 87.3% (90/103) of the participants in the control group provided pain and function primary outcomes, respectively.

**Table 1:** Baseline descriptive characteristics by treatment group.

Baseline variable	Intervention group (n=103)	Control group (n=103)
Age (years), mean (SD)	60.3 (8.2)	59.0 (8.5)
Female, n (%)	60 (58.2)	66 (64.1)
Number of comorbidities^a^, mean (SD)	0.8 (1)	0.8 (0.9)
Number of other joints with pain^b^, mean (SD)	1.7 (1.5)	1.9 (1.7)
Arthritis self-efficacy pain subscale^c^, mean (SD)	6.0 (1.7)	6.0 (1.7)
Self-efficacy for exercise^d^, mean (SD)	60.6 (21.5)	58.8 (18.6)
Exercise importance^e^, mean (SD)	6.1 (1.2)	6.1 (1.2)

^a^Collected via a question asking participants to select from a list of 13 comorbidities any of which were relevant to them. A participant’s selected comorbidities were then added to create a continuous score of the total number of comorbidities per participant; this resulted in a range of 0 to 4.

^b^Collected via a question asking participants to select from a list of 9 joints any of which they currently experience pain in. Responses were converted into a continuous score of number of other joints with pain per participant, ranging from 0 to 9.

^c^Scores range from 1 to 10, with higher scores indicating greater self-efficacy for pain.

^d^Scores range from 0 to 90, with higher scores indicating greater self-efficacy for exercise.

^e^Measured via the response to the question “How important is it to you to do regular exercise to manage your knee condition?” Scores range from 1 to 7; higher score indicates higher importance.

### Moderators of the Effect of the Intervention on Change in Physical Function

**Figure 1:**
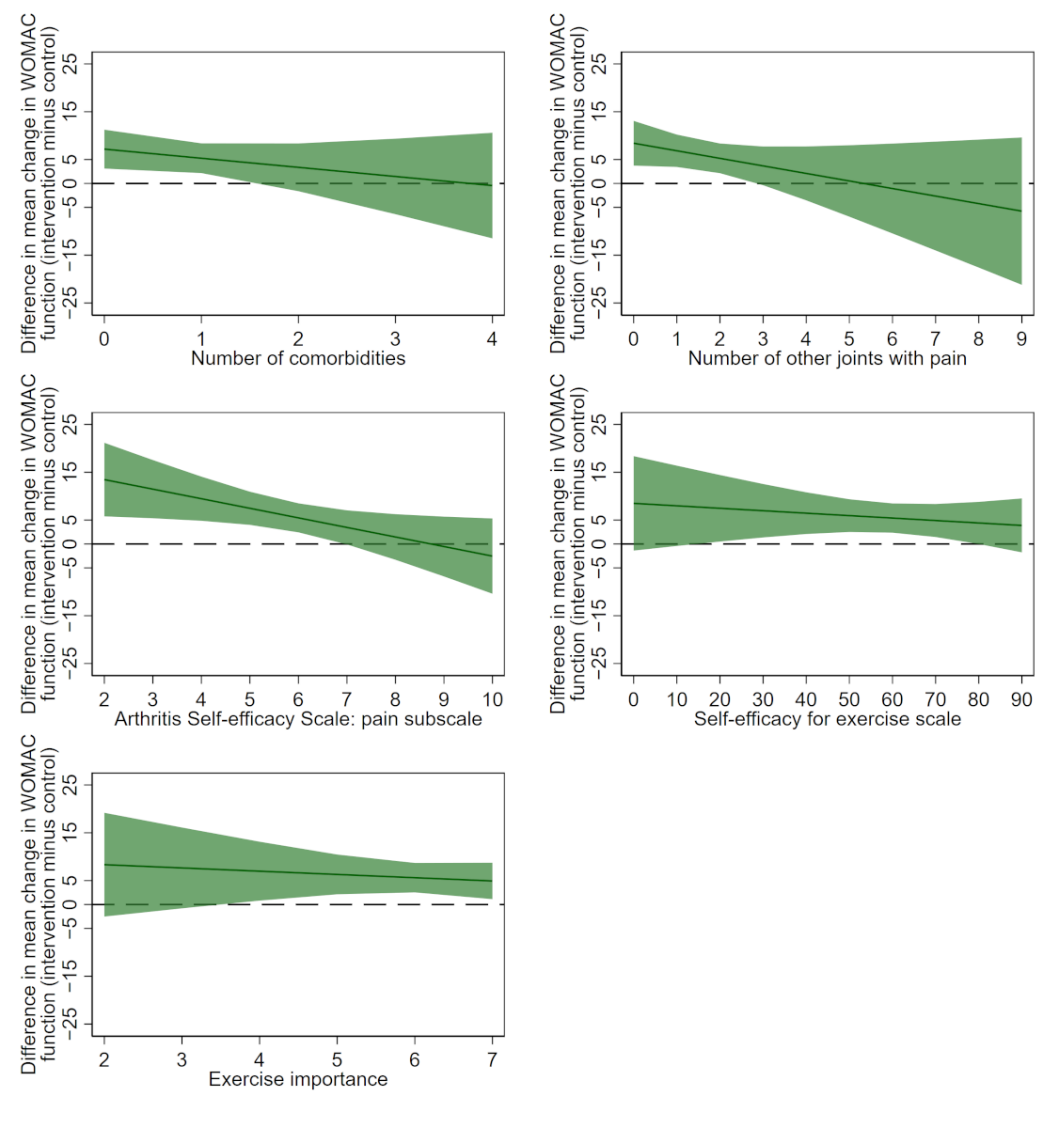
Differences in the mean change in Western Ontario and McMaster Universities Osteoarthritis Index function (baseline minus 24 weeks) between treatment groups (intervention minus control) for each potential continuous moderator by using multiply imputed data. Positive values favor the intervention. The solid line indicates the difference between the control and intervention arms. Dashed line indicates no difference between the control and intervention arms. Shaded areas indicate 95% confidence intervals. WOMAC: Western Ontario and McMaster Universities Osteoarthritis Index.

**Figure 2:**
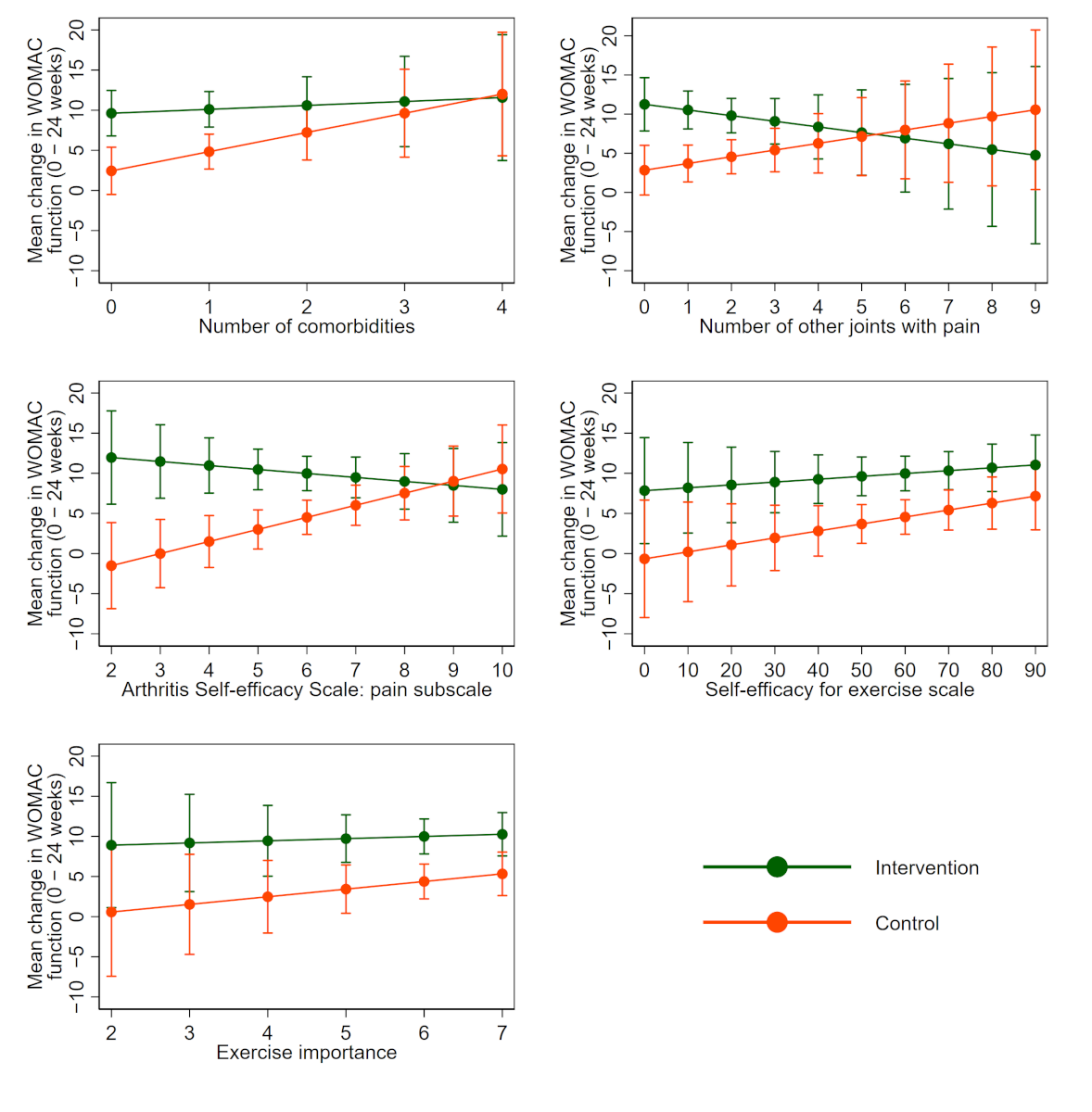
Mean change in Western Ontario and McMaster Universities Osteoarthritis Index function (baseline minus 24 weeks) in each treatment group for each potential continuous moderator by using multiply imputed data. Positive changes indicate improvement. The solid line indicates the average change in each treatment group. Bars indicate 95% confidence intervals. WOMAC: Western Ontario and McMaster Universities Osteoarthritis Index.

Findings from the fractional polynomial assessment indicated that the models assuming a linear relationship between each potential moderator and change in physical function provided the best fit; therefore, more complex models were not needed (refer to scatter plots in Multimedia Appendix 2). Results of linear models using multiply imputed data are presented in Table 2 and visually in Figure 1 and Figure 2. There was evidence that pain self-efficacy moderated the effect of the intervention on physical function compared to the control at 24 weeks using multiply imputed data (estimated mean difference –2.14, 95% CI –3.96 to –0.33; *P*=.02). Posthoc assessment of the mean change in WOMAC function by treatment arm following identification of an interaction effect showed that each 1-unit increase in baseline pain self-efficacy was associated with a 1.52 (95% CI 0.27 to 2.78) WOMAC units improvement in the control group. In contrast, with each unit increase in pain self-efficacy, a reduction of 0.62 (95% CI –1.93 to 0.68) WOMAC units was observed in the intervention group. There was only weak evidence that the other selected baseline variables moderated the effect of the intervention on physical function compared to the control at 24 weeks (Table 2). Additionally, results show positive associations between each number of comorbidities, self-efficacy for exercise and exercise importance, and change in WOMAC function for both control and intervention groups (Figure 2). There appears to be a negative relationship between number of other joints with pain and change in WOMAC function for the intervention group but a positive relationship for the control group (Figure 2). Results using complete case data were similar (Multimedia Appendix 3 and Multimedia Appendix 4).

**Table 2:** Results of the moderation analysis presented in terms of the effect on change in Western Ontario and McMaster Universities Osteoarthritis Index function of a 1-unit increase in the potential moderators in each of the control and intervention groups using multiply imputed data.

Moderator (taken at baseline)	Estimated moderator coefficient (95% CI)			Interaction *P* value
	Intervention group	Control group		
Number of comorbidities^a^	0.49 (–1.68 to 2.66)	2.41 (0.01 to 4.81)	.24	
Number of other joints with pain^b^	–0.68 (–2.21 to 0.84)	0.76 (–0.55 to 2.07)	.16	
Arthritis self-efficacy pain subscale^c^	–0.62 (–1.93 to 0.68)	1.52 (0.27 to 2.78)	.02	
Self-efficacy for exercise scale^d^	0.04 (–0.07 to 0.14)	0.09 (–0.03 to 0.20)	.54	
Exercise importance^e^	0.33 (–1.48 to 2.13)	1.00 (–0.81 to 2.81)	.61	

^a^Collected via a question asking participants to select from a list of 13 comorbidities any of which were relevant to them. A participant’s selected comorbidities were then added to create a continuous score of the total number of comorbidities per participant; this resulted in a range of 0 to 4.

^b^Collected via a question asking participants to select from a list of 9 joints any of which they currently experience pain in. Responses were converted into a continuous score of number of other joints with pain per participant, ranging from 0 to 9.

^c^Scores range from 1 to 10, with higher scores indicating greater self-efficacy for pain.

^d^Scores range from 0 to 90, with higher scores indicating greater self-efficacy for exercise.

^e^Measured via the response to the question “How important is it to you to do regular exercise to manage your knee condition?” Scores range from 1 to 7; higher score indicates higher importance.

### Moderators of the Effect of the Intervention on Change in Overall Pain

**Figure 3:**
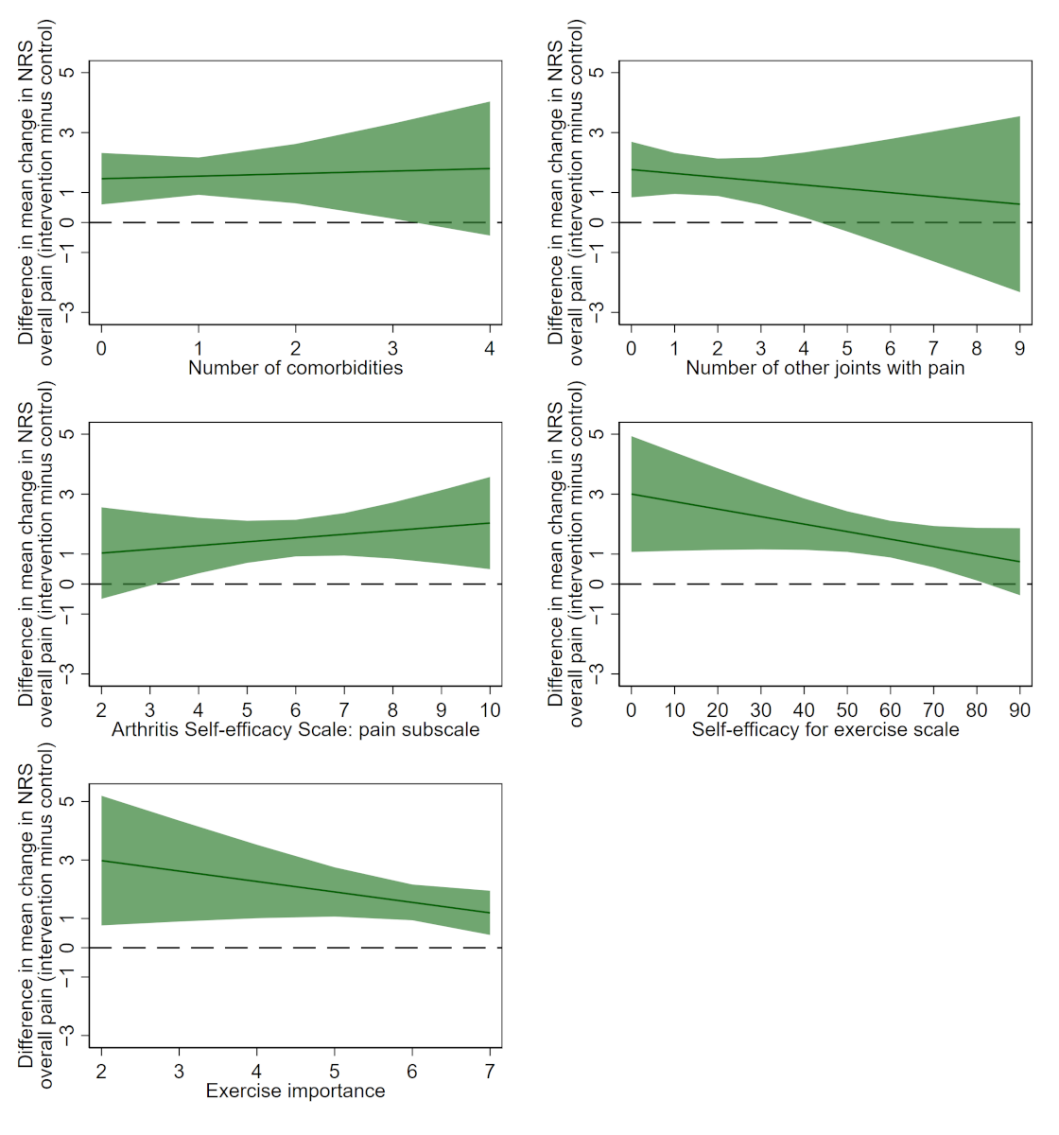
Differences in the mean change in the numerical rating scale for overall knee pain (baseline minus 24 weeks) between treatment groups (intervention minus control) for each potential continuous moderator by using multiply imputed data. Positive values favor the intervention. The solid line indicates the difference between the control and intervention arms. The dashed line indicates no difference between the control and intervention arms. Shaded areas indicate 95% confidence intervals. NRS: numerical rating scale.

**Figure 4:**
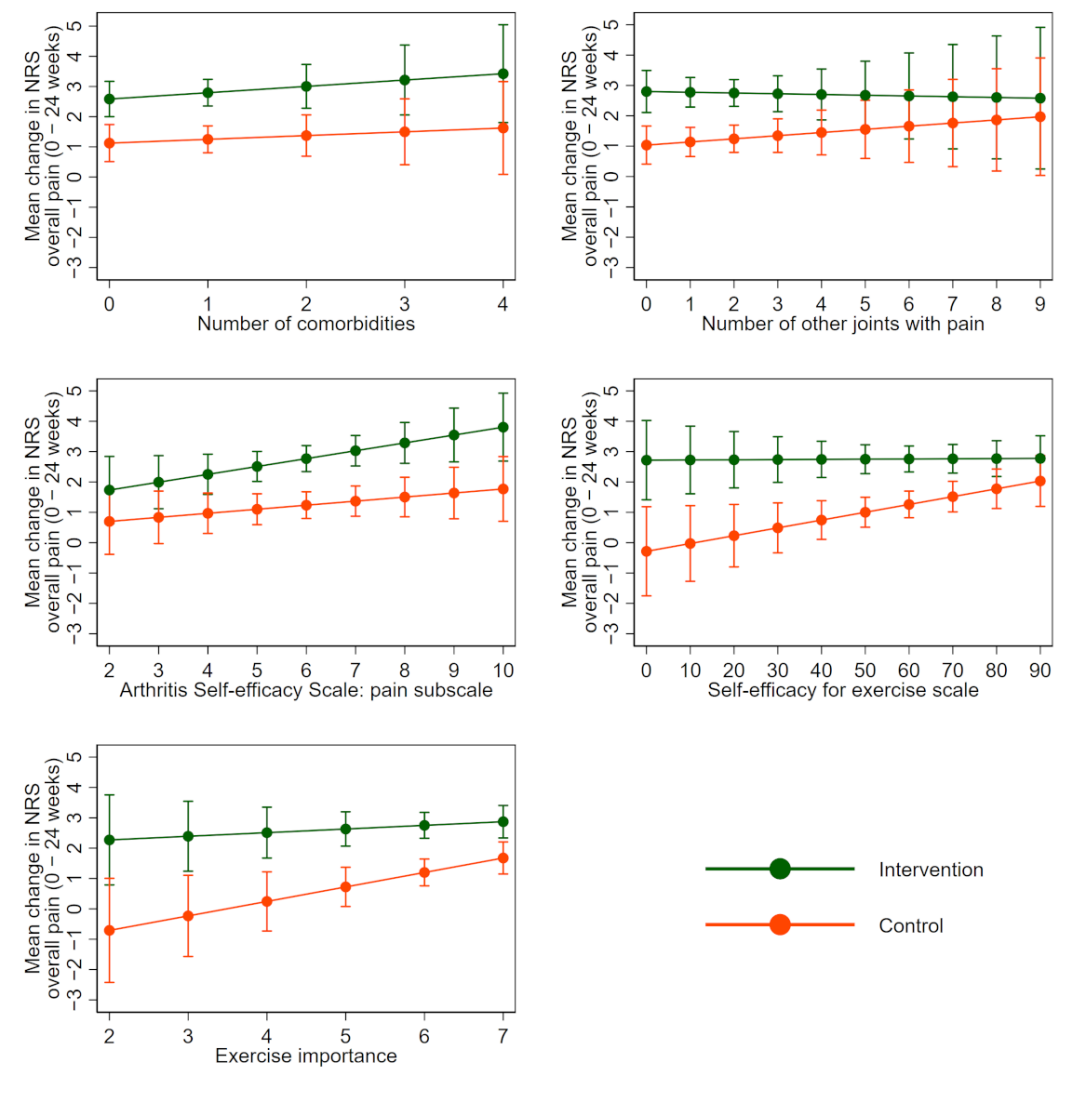
Mean change in the numerical rating scale for overall knee pain (baseline minus 24 weeks) in each treatment group for each potential continuous moderator by using multiply imputed data. Positive changes indicate improvement. The solid line indicates the average change in each treatment group. Bars indicate 95% confidence intervals. NRS: numerical rating scale.

The models assuming a linear relationship between each potential moderator and change in overall pain provided the best fit; therefore, more complex models were not needed (refer to scatter plots in Multimedia Appendix 5). The results of linear models using multiply imputed data are presented in Table 3 and visually in Figure 3 and Figure 4. There was only weak evidence that any of the investigated variables moderated the effect of the intervention on pain compared to the control at 24 weeks. In general, positive relationships were observed between each of the baseline characteristics and change in overall pain for both intervention and control arms, with little difference in the magnitude of the slope for each group (Figure 4). Results using complete case data were similar (Multimedia Appendix 3 and Multimedia Appendix 6).

**Table 3:** Results of the moderation analysis, presented in terms of the effect on change in the numerical rating scale of overall knee pain of a 1-unit increase in the potential moderators in each of the control and intervention groups using multiply imputed data.

Moderator (taken at baseline)	Estimated moderator coefficient (95% CI)			Interaction *P* value
	Intervention group	Control group		
Number of comorbidities^a^	0.23 (–0.21 to 0.68)	0.13 (–0.35 to 0.62)	.76	
Number of other joints with pain^b^	–0.02 (–0.32 to 0.28)	0.10 (–0.16 to 0.36)	.56	
Arthritis self-efficacy pain subscale^c^	0.23 (–0.02 to 0.49)	0.14 (–0.10 to 0.38)	.60	
Self-efficacy for exercise scale^d^	0.00 (–0.02 to 0.02)	0.02 (0.00 to 0.05)	.13	
Exercise importance^e^	0.11 (–0.23 to 0.45)	0.47 (0.12 to 0.83)	.15	

^a^Collected via a question asking participants to select from a list of 13 comorbidities any of which were relevant to them. A participant’s selected comorbidities were then added to create a continuous score of the total number of comorbidities per participant; this resulted in a range of 0 to 4.

^b^Collected via a question asking participants to select from a list of 9 joints, any of which they currently experience pain in. Responses were converted into a continuous score of number of other joints with pain per participant, ranging from 0 to 9.

^c^Scores range from 1 to 10, with higher scores indicating greater self-efficacy for pain.

^d^Scores range from 0 to 90, with higher scores indicating greater self-efficacy for exercise.

^e^Measured via the response to the question “How important is it to you to do regular exercise to manage your knee condition?” Scores range from 1 to 7; higher scores indicate higher importance.

## Discussion

### Principal Findings

This study explored potential moderators of the effect of a self-directed digitally delivered exercise intervention for people with knee OA on changes in pain and physical function over 24 weeks. Except for pain self-efficacy, we found little evidence that our selected patient characteristics moderated treatment outcomes. Regarding pain self-efficacy, the hypothesis generated from this exploratory study is that participants with higher pain self-efficacy at baseline experienced smaller improvements in function with the self-directed digitally delivered exercise intervention compared to the control. Conversely, participants with lower pain self-efficacy at baseline experienced greater improvements in function from the intervention compared to the control. After identifying an interaction effect between pain self-efficacy and function, we further explored our finding by treatment group. We identified that the interaction effect was driven by changes in the control group. Specifically, each unit increase in pain self-efficacy was associated with a 1.52-unit (95% CI 0.27 to 2.78) improvement in function in the control group. In contrast, there was a reduction in function of 0.62 units (95% CI –1.93 to 0.68) for each unit increase in pain self-efficacy in the intervention group, although this finding is equivocal, given the confidence interval crossed zero. Pain self-efficacy refers to one’s confidence in their ability to control their pain and function while in pain [30] and determines how much effort people will expend and how long they will persist while experiencing pain [33]. Therefore, it may be possible that participants in the control group who had higher baseline pain self-efficacy were more confident and may have felt more willing to act upon the general knee exercise information offered in the control website while experiencing pain. Hence, this may be why people in the control group with greater baseline pain self-efficacy (over 4/10) experienced improvements in function while those with lower pain self-efficacy (less than 4/10) experienced worsening function. This suggests that for people with knee OA and low pain self-efficacy, interventions that combine exercise with strategies to improve pain self-efficacy such as evidence-based pain education and psychological interventions (eg, pain coping skills training) [34] may be more appropriate than education only.

There is limited research investigating the moderating effects of self-efficacy on exercise outcomes in knee OA. One exploratory study found evidence that pain self-efficacy moderated the effect of a telehealth-delivered physiotherapist-prescribed exercise program combined with an automated pain coping skills training program for people with knee OA on changes in pain but not function when compared to an education control [25]. This contrasts our findings where pain self-efficacy moderated changes in function but not pain. The reason for the variation in the findings between the 2 studies is unclear; however, it may relate to the vast differences between the interventions. For example, the former study included physiotherapist-prescribed exercise and psychological treatment addressing pain coping, while the intervention in our study comprised fully self-directed exercise and automated exercise adherence support. Even so, these differences in findings highlight the need for future, adequately powered studies to rule out chance findings and rigorously explore the moderating role of pain self-efficacy on exercise effects and the related mechanisms that may be at play in people with knee OA. Regarding self-efficacy for exercise, one study has conducted a secondary analysis of data from an RCT comparing exercise, self-management, and active coping strategies (individual-delivered and group-delivered) to usual care on function in people with chronic knee pain [35]. Similar to our findings, this study found no evidence that baseline self-efficacy for exercise (measured in this study via a subscale of an exercise health beliefs and self-efficacy questionnaire) moderated the effect of the intervention on function at 6 months compared to the usual care control. A possible explanation for these findings may relate to uncertainties about the effect of exercise adherence on knee OA outcomes. For example, although self-efficacy for exercise is associated with higher levels of physical activity/exercise participation in people with knee pain [26], greater exercise adherence may not actually translate to improved knee OA outcomes [36]. As these findings are all exploratory in nature, further studies are required to explore the potential of self-efficacy to moderate the effects of exercise on OA symptoms. Nonetheless, our findings do demonstrate that patients with all levels of self-efficacy experienced improvements in function and reductions in knee pain from the self-directed digitally delivered exercise intervention, which supports the use of this intervention for people with knee OA and all levels of self-efficacy at baseline.

To our knowledge, this is the first study to investigate multi-joint pain and comorbidities as moderators of the effect of self-directed exercise on outcomes in people with OA. We found that neither of these baseline characteristics moderated the effect of the intervention on changes in pain or function relative to the control. Despite this, an interesting observation was a negative relationship between number of other joints with pain and changes in physical function for the intervention group (ie, those with a greater number of joints with pain experienced smaller improvements in function). Although, this was supported by only weak evidence of an interaction effect (*P*=.16), this observation may indicate that the self-directed digitally delivered intervention may be less beneficial for people with pain in multiple joints. Multi-joint pain can be indicative of complex pain presentations such as widespread pain and fibromyalgia [37], and people with knee OA and widespread pain have been found to have poorer self-reported function when compared to people with knee OA only [27,38]. Furthermore, personalized exercise prescription and monitoring (eg, via a health professional) and psychological treatments such as cognitive behavior therapy are recommended for people with widespread pain [39]. Therefore, people with knee OA and pain in multiple other joints may be better suited to a tailored approach to management over a self-directed digitally delivered exercise intervention. Regarding comorbidity, 2 studies have explored the potential for the number of comorbidities present at baseline to moderate the effect of therapist-led exercise on pain and function in people with knee OA and support our findings [40,41]. A systematic review of subgroup analyses from 14 RCTs found that the number of comorbidities present at baseline did not moderate the effects of exercise interventions on pain or function compared to nonexercise controls for people with knee or hip OA [40]. Similarly, a recent secondary analysis of RCT data found that the number of comorbidities present at baseline did not moderate the effect of a course of physiotherapist-led exercise on pain and function at 6 months compared with a nonexercise control [41]. Although these findings are exploratory and require confirmation in future studies, they do indicate that people with knee OA and multiple comorbid conditions may respond to exercise in similar ways as those without comorbidity. This may be unsurprising, considering exercise is safe and effective for people with multimorbidity and is recommended for a broad range of chronic conditions [42].

There appears to be currently no research exploring patient characteristics as potential moderators of the effects of digitally delivered exercise in populations with OA and limited research in adult populations more broadly. We found 2 studies that conducted subgroup analyses of RCT data exploring potential moderators of the effect of digitally delivered physical activity interventions in adults. One study explored potential moderators of the effect of computer-tailored physical activity on changes in total weekly minutes of physical activity at 12 months in people aged 50 years and older [43]. Moderation analysis found participants with a higher age, lower body mass index, and higher self-reported intention to be physically active at baseline were not responsive to computer-tailored physical activity relative to a waitlist control. Another study explored potential moderators of the effect of computer-tailored physical activity intervention on changes in physical activity behavior (accelerometry measured moderate-to-vigorous physical activity per week and steps per day) at 3 months in inactive adults [44]. This study found that the intervention was more effective for women than men, relative to the usual care control. Owing to the differences in the selected moderators and outcomes, direct comparisons cannot be made with our findings. However, our findings add to the scant literature regarding the role of patient characteristics as moderators of the effect of digitally delivered exercise in adult populations and may provide direction for the selection of potential moderators in future research.

Overall, our findings suggest that our self-directed digitally delivered exercise intervention is similarly effective for pain for a range of people with knee OA. With respect to function, it may be that our 24-week intervention is less beneficial when compared to control for people with high baseline pain self-efficacy (eg, a score of over 9/10; see Figure 2). Conversely, the intervention may be more beneficial when compared to control for people with lower levels of pain self-efficacy. Therefore, it may be a novel treatment approach for people with knee OA and low pain self-efficacy who typically report greater levels of disability [45].

### Strengths and Limitations

The strengths of this study include its robust design using reliable and valid clinical outcome measures [22-24] and appropriate methods of statistical analyses of interaction/moderation [18]. Further, our selection of potential moderator variables was evidence-informed and explicitly described *a priori* while the selection of only a few variables aimed to minimize the risk of potentially erroneous findings. Our findings and the hypothesis generated can also be considered generalizable to the broader knee OA population owing to the limited inclusion criteria and nationwide community recruitment of participants in the RCT. Several limitations must also be acknowledged. This study was exploratory as the original RCT was powered to detect changes in pain and function and not to detect moderator effects. As this was exploratory, no adjustment for multiple testing was conducted. It is possible that a lack of power prevented the identification of potential moderators rather than the absence of an effect or that our finding occurred by chance, although a chance finding was controlled for by limiting the number of potential moderators to analyze. As such, further confirmatory studies are required. Additionally, despite our selection of variables being evidence-informed, it is possible that baseline variables other than those selected could be potential moderators.

### Conclusion

With the exception of pain self-efficacy, we found little evidence that our selected patient characteristics moderated treatment outcomes. Although these findings are exploratory in nature, they do contribute to the sparse literature regarding moderators of the effect of digitally delivered exercise in adult populations and may inform future research aiming to improve the targeting of exercise treatments.

## Appendix

Multimedia Appendix 1 Overview of the selected moderators and their rationale for inclusion.

Multimedia Appendix 2 Change in function (baseline minus 24 weeks) against each continuous potential moderator by treatment group by using multiply imputed data.

Multimedia Appendix 3 Results of the moderation analysis, presented in terms of the effect on the primary outcomes of a 1-unit increase in the moderators in each of the control and intervention groups by using complete case data.

Multimedia Appendix 4 Difference in mean change in Western Ontario and McMaster Universities Osteoarthritis Index function (baseline minus 24 weeks) between treatment groups (intervention minus control) for each potential continuous moderator by using complete case data.

Multimedia Appendix 5 Change in overall pain (baseline minus 24 weeks) against each continuous potential moderator, by treatment group using multiply imputed data.

Multimedia Appendix 6 Difference in mean change in overall knee pain (baseline minus 24 weeks) between treatment groups (intervention minus control) for each potential continuous moderator by using complete case data.

## Abbreviations

OA: osteoarthritis
RCT: randomized controlled trial
WOMAC: Western Ontario and McMaster Universities Osteoarthritis Index

## References

[1] Nuki G (1999). Osteoarthritis: a problem of joint failure. Z Rheumatol.

[2] Pereira D, Peleteiro B, Araújo J, Branco J, Santos RA, Ramos E (2011). The effect of osteoarthritis definition on prevalence and incidence estimates: a systematic review. Osteoarthritis Cartilage.

[3] Dieppe P, Cushnaghan J, Tucker M, Browning S, Shepstone L (2000). The Bristol 'OA500 study': progression and impact of the disease after 8 years. Osteoarthritis Cartilage.

[4] Sharma L, Cahue S, Song J, Hayes K, Pai Y, Dunlop D (2003). Physical functioning over three years in knee osteoarthritis: role of psychosocial, local mechanical, and neuromuscular factors. Arthritis Rheum.

[5] Safiri S, Kolahi A, Smith E, Hill C, Bettampadi D, Mansournia MA, Hoy D, Ashrafi-Asgarabad A, Sepidarkish M, Almasi-Hashiani A, Collins G, Kaufman J, Qorbani M, Moradi-Lakeh M, Woolf AD, Guillemin F, March L, Cross M (2020). Global, regional and national burden of osteoarthritis 1990-2017: a systematic analysis of the Global Burden of Disease Study 2017. Ann Rheum Dis.

[6] Kolasinski SL, Neogi T, Hochberg MC, Oatis C, Guyatt G, Block J, Callahan L, Copenhaver C, Dodge C, Felson D, Gellar K, Harvey WF, Hawker G, Herzig E, Kwoh CK, Nelson AE, Samuels J, Scanzello C, White D, Wise B, Altman RD, DiRenzo D, Fontanarosa J, Giradi G, Ishimori M, Misra D, Shah AA, Shmagel AK, Thoma LM, Turgunbaev M, Turner AS, Reston J (2020). 2019 American College of Rheumatology/Arthritis Foundation Guideline for the Management of Osteoarthritis of the Hand, Hip, and Knee. Arthritis Rheumatol.

[7] Bannuru RR, Osani MC, Vaysbrot EE, Arden NK, Bennell K, Bierma-Zeinstra SMA, Kraus VB, Lohmander LS, Abbott JH, Bhandari M, Blanco FJ, Espinosa R, Haugen IK, Lin J, Mandl LA, Moilanen E, Nakamura N, Snyder-Mackler L, Trojian T, Underwood M, McAlindon TE (2019). OARSI guidelines for the non-surgical management of knee, hip, and polyarticular osteoarthritis. Osteoarthritis Cartilage.

[8] The Royal Australian College of General Practitioners (2018). Guideline for the Management of Knee and Hip Osteoarthritis, Second edition.

[9] (2020). Osteoarthritis: care and management. National Institute for Health Clinical Excellence Clinical Guideline No. 177.

[10] Fransen M, McConnell S, Harmer AR, Van der Esch M, Simic M, Bennell KL (2015). Exercise for osteoarthritis of the knee: a Cochrane systematic review. Br J Sports Med.

[11] Holden MA, Burke DL, Runhaar J, van Der Windt D, Riley RD, Dziedzic K, Legha A, Evans AL, Abbott JH, Baker K, Brown J, Bennell KL, Bossen D, Brosseau L, Chaipinyo K, Christensen R, Cochrane T, de Rooij M, Doherty M, French HP, Hickson S, Hinman RS, Hopman-Rock M, Hurley MV, Ingram C, Knoop J, Krauss I, McCarthy C, Messier SP, Patrick DL, Sahin N, Talbot LA, Taylor R, Teirlinck CH, van Middelkoop M, Walker C, Foster NE, Trial Bank Oa (2017). Subgrouping and TargetEd Exercise pRogrammes for knee and hip OsteoArthritis (STEER OA): a systematic review update and individual participant data meta-analysis protocol. BMJ Open.

[12] Kraemer HC, Wilson GT, Fairburn CG, Agras WS (2002). Mediators and moderators of treatment effects in randomized clinical trials. Arch Gen Psychiatry.

[13] Fernandes L, Hagen KB, Bijlsma JWJ, Andreassen O, Christensen P, Conaghan PG, Doherty M, Geenen R, Hammond A, Kjeken I, Lohmander LS, Lund H, Mallen CD, Nava T, Oliver S, Pavelka K, Pitsillidou I, da Silva José Antonio, de la Torre Jenny, Zanoli G, Vliet Vlieland Theodora P M, European League Against Rheumatism (EULAR) (2013). EULAR recommendations for the non-pharmacological core management of hip and knee osteoarthritis. Ann Rheum Dis.

[14] Kvedar JC, Fogel AL, Elenko E, Zohar D (2016). Digital medicine's march on chronic disease. Nat Biotechnol.

[15] Nelligan RK, Hinman RS, Kasza J, Crofts SJC, Bennell KL (2021). Effects of a Self-directed Web-Based Strengthening Exercise and Physical Activity Program Supported by Automated Text Messages for People With Knee Osteoarthritis: A Randomized Clinical Trial. JAMA Intern Med.

[16] Bossen D, Buskermolen M, Veenhof C, de Bakker Dinny, Dekker J (2013). Adherence to a web-based physical activity intervention for patients with knee and/or hip osteoarthritis: a mixed method study. J Med Internet Res.

[17] Baruth M, Wilcox S, Sharpe P, Schoffman D, Becofsky K (2014). Baseline predictors of physical activity in a sample of adults with arthritis participating in a self-directed exercise program. Public Health.

[18] Royston P, Sauerbrei W (2009). Two Techniques for Investigating Interactions between Treatment and Continuous Covariates in Clinical Trials. The Stata Journal.

[19] Nelligan RK, Hinman RS, Kasza J, Bennell KL (2019). Effectiveness of internet-delivered education and home exercise supported by behaviour change SMS on pain and function for people with knee osteoarthritis: a randomised controlled trial protocol. BMC Musculoskelet Disord.

[20] Nelligan RK, Hinman RS, Atkins L, Bennell KL (2019). A Short Message Service Intervention to Support Adherence to Home-Based Strengthening Exercise for People With Knee Osteoarthritis: Intervention Design Applying the Behavior Change Wheel. JMIR Mhealth Uhealth.

[21] Michie S, Atkins L, West R (2014). The Behaviour Change Wheel: A Guide To Designing Interventions.

[22] McAlindon TE, Driban JB, Henrotin Y, Hunter DJ, Jiang G, Skou ST, Wang S, Schnitzer T (2015). OARSI Clinical Trials Recommendations: Design, conduct, and reporting of clinical trials for knee osteoarthritis. Osteoarthritis Cartilage.

[23] Bellamy N, Buchanan WW, Goldsmith CH, Campbell J, Stitt LW (1988). Validation study of WOMAC: a health status instrument for measuring clinically important patient relevant outcomes to antirheumatic drug therapy in patients with osteoarthritis of the hip or knee. J Rheumatol.

[24] Rolfson O, Wissig S, van Maasakkers L, Stowell C, Ackerman I, Ayers D, Barber T, Benzakour T, Bozic K, Budhiparama N, Caillouette J, Conaghan PG, Dahlberg L, Dunn J, Grady-Benson J, Ibrahim SA, Lewis S, Malchau H, Manzary M, March L, Nassif N, Nelissen R, Smith N, Franklin PD (2016). Defining an International Standard Set of Outcome Measures for Patients With Hip or Knee Osteoarthritis: Consensus of the International Consortium for Health Outcomes Measurement Hip and Knee Osteoarthritis Working Group. Arthritis Care Res (Hoboken).

[25] Lawford BJ, Hinman RS, Kasza J, Nelligan R, Keefe F, Rini C, Bennell KL (2018). Moderators of Effects of Internet-Delivered Exercise and Pain Coping Skills Training for People With Knee Osteoarthritis: Exploratory Analysis of the IMPACT Randomized Controlled Trial. J Med Internet Res.

[26] Quicke JG, Foster NE, Ogollah RO, Croft PR, Holden MA (2017). Relationship Between Attitudes and Beliefs and Physical Activity in Older Adults With Knee Pain: Secondary Analysis of a Randomized Controlled Trial. Arthritis Care Res (Hoboken).

[27] Bergman S, Thorstensson C, Andersson MLE (2019). Chronic widespread pain and its associations with quality of life and function at a 20- year follow-up of individuals with chronic knee pain at inclusion. BMC Musculoskelet Disord.

[28] Mahgoub MY, Elnady BM, et al (2020). Erratum: Comorbidity of Fibromyalgia in Primary Knee Osteoarthritis: Potential Impact on Functional Status and Quality of Life [Corrigendum]. Open Access Rheumatol.

[29] Dobson Fiona, Bennell Kim L, French Simon D, Nicolson Philippa J A, Klaasman Remco N, Holden Melanie A, Atkins Lou, Hinman Rana S (2016). Barriers and Facilitators to Exercise Participation in People with Hip and/or Knee Osteoarthritis: Synthesis of the Literature Using Behavior Change Theory. Am J Phys Med Rehabil.

[30] Nicholas MK (2007). The pain self-efficacy questionnaire: Taking pain into account. Eur J Pain.

[31] Lorig K, Chastain RL, Ung E, Shoor S, Holman HR (1989). Development and evaluation of a scale to measure perceived self-efficacy in people with arthritis. Arthritis Rheum.

[32] Resnick B, Jenkins LS (2000). Testing the reliability and validity of the Self-Efficacy for Exercise scale. Nurs Res.

[33] Bandura A, Freeman WH, Lightsey R (1999). Self-Efficacy: The Exercise of Control. J Cogn Psychother.

[34] Keefe F, Caldwell D, Williams D, Gil K, Mitchell D, Robertson C (1990). Pain coping skills training in the management of osteoarthritic knee pain: A comparative study. Behavior Therapy.

[35] Hurley MV, Walsh NE, Mitchell HL, Pimm TJ, Patel A, Williamson E, Jones RH, Dieppe PA, Reeves BC (2007). Clinical effectiveness of a rehabilitation program integrating exercise, self-management, and active coping strategies for chronic knee pain: a cluster randomized trial. Arthritis Rheum.

[36] Nicolson PJ, Hinman RS, Wrigley TV, Stratford PW, Bennell KL (2019). Effects of Covertly Measured Home Exercise Adherence on Patient Outcomes Among Older Adults With Chronic Knee Pain. J Orthop Sports Phys Ther.

[37] Wolfe F, Smythe HA, Yunus MB, Bennett RM, Bombardier C, Goldenberg DL, Tugwell P, Campbell SM, Abeles M, Clark P (1990). The American College of Rheumatology 1990 Criteria for the Classification of Fibromyalgia. Report of the Multicenter Criteria Committee. Arthritis Rheum.

[38] Guérard Olivier, Dufort S, Forget Besnard L, Gougeon A, Carlesso L (2020). Comparing the association of widespread pain, multi-joint pain and low back pain with measures of pain sensitization and function in people with knee osteoarthritis. Clin Rheumatol.

[39] Macfarlane GJ, Kronisch C, Dean LE, Atzeni F, Häuser W, Fluß E, Choy E, Kosek E, Amris K, Branco J, Dincer F, Leino-Arjas P, Longley K, McCarthy GM, Makri S, Perrot S, Sarzi-Puttini P, Taylor A, Jones GT (2017). EULAR revised recommendations for the management of fibromyalgia. Ann Rheum Dis.

[40] Quicke J, Runhaar J, van der Windt D, Healey E, Foster N, Holden M (2020). Moderators of the effects of therapeutic exercise for people with knee and hip osteoarthritis: A systematic review of sub-group analyses from randomised controlled trials. Osteoarthritis and Cartilage Open.

[41] Legha A, Burke DL, Foster NE, van der Windt DA, Quicke JG, Healey EL, Runhaar J, Holden MA (2020). Do comorbidities predict pain and function in knee osteoarthritis following an exercise intervention, and do they moderate the effect of exercise? Analyses of data from three randomized controlled trials. Musculoskeletal Care.

[42] Bricca A, Harris L, Jäger Madalina, Smith S, Juhl C, Skou S (2020). Benefits and harms of exercise therapy in people with multimorbidity: A systematic review and meta-analysis of randomised controlled trials. Ageing Res Rev.

[43] van Stralen MM, de Vries H, Bolman C, Mudde AN, Lechner L (2010). Exploring the efficacy and moderators of two computer-tailored physical activity interventions for older adults: a randomized controlled trial. Ann Behav Med.

[44] To Q, Duncan M, Short C, Plotnikoff R, Kerry Mummery W, Alley S, Schoeppe Stephanie, Rebar Amanda, Vandelanotte Corneel (2021). Examining moderators of the effectiveness of a web- and video-based computer-tailored physical activity intervention. Prev Med Rep.

[45] Marks R (2014). Self-efficacy and arthritis disability: An updated synthesis of the evidence base and its relevance to optimal patient care. Health Psychol Open.

