# Supplementary material for: Moderators of the Effect of a Self-directed Digitally Delivered Exercise Program for People With Knee Osteoarthritis: Exploratory Analysis of a Randomized Controlled Trial
Source: J Med Internet Res. 2021 Oct 29;23(10):e30768. doi: 10.2196/30768 (PMC8590189; doi:10.2196/30768)
Supplement: Multimedia Appendix 1 [file jmir_v23i10e30768_app1.pdf]

Multimedia Appendix 1: Overview of selected moderators and their rationale for inclusion

Baseline variable	Rationale
Number of comorbidities	Based on evidence that comorbidities are associated with usage of a self-directed web-based physical activity program [16] and with physical activity behaviour post self-directed exercise [17].
Number of other joints with pain	Chosen based on theoretical rationale that multi-joint pain can be indicative of additional pain syndromes which are commonly observed in knee OA populations [36, 44]. People with knee OA and pain in additional joints may require more comprehensive management than a self-directed digitally-delivered exercise intervention.
Arthritis Self Efficacy Scale: pain subscale	Based on evidence that pain self-efficacy is a potential moderator of the effect of internet delivered physiotherapist-prescribed exercise and pain coping skills training on pain, in people with knee OA [30].
Self-efficacy for Exercise	Based on evidence that self-efficacy for exercise is associated with physical activity behaviour post 1:1 physical therapist–led exercise [32].
Exercise importance	Chosen based on theoretical rationale. There is evidence to suggest positive views of exercise can facilitates exercise participation [45] and predict exercise behaviour [32] in people with knee OA, which may optimise exercise outcomes.
