# Supplementary material for: Moderators of the Effect of a Self-directed Digitally Delivered Exercise Program for People With Knee Osteoarthritis: Exploratory Analysis of a Randomized Controlled Trial
Source: J Med Internet Res. 2021 Oct 29;23(10):e30768. doi: 10.2196/30768 (PMC8590189; doi:10.2196/30768)

Multimedia Appendix 2: Change in WOMAC (Western Ontario and McMaster Universities Osteoarthritis Index) function (baseline minus 24 weeks) against each continuous potential moderator, by treatment group using multiply imputed data.

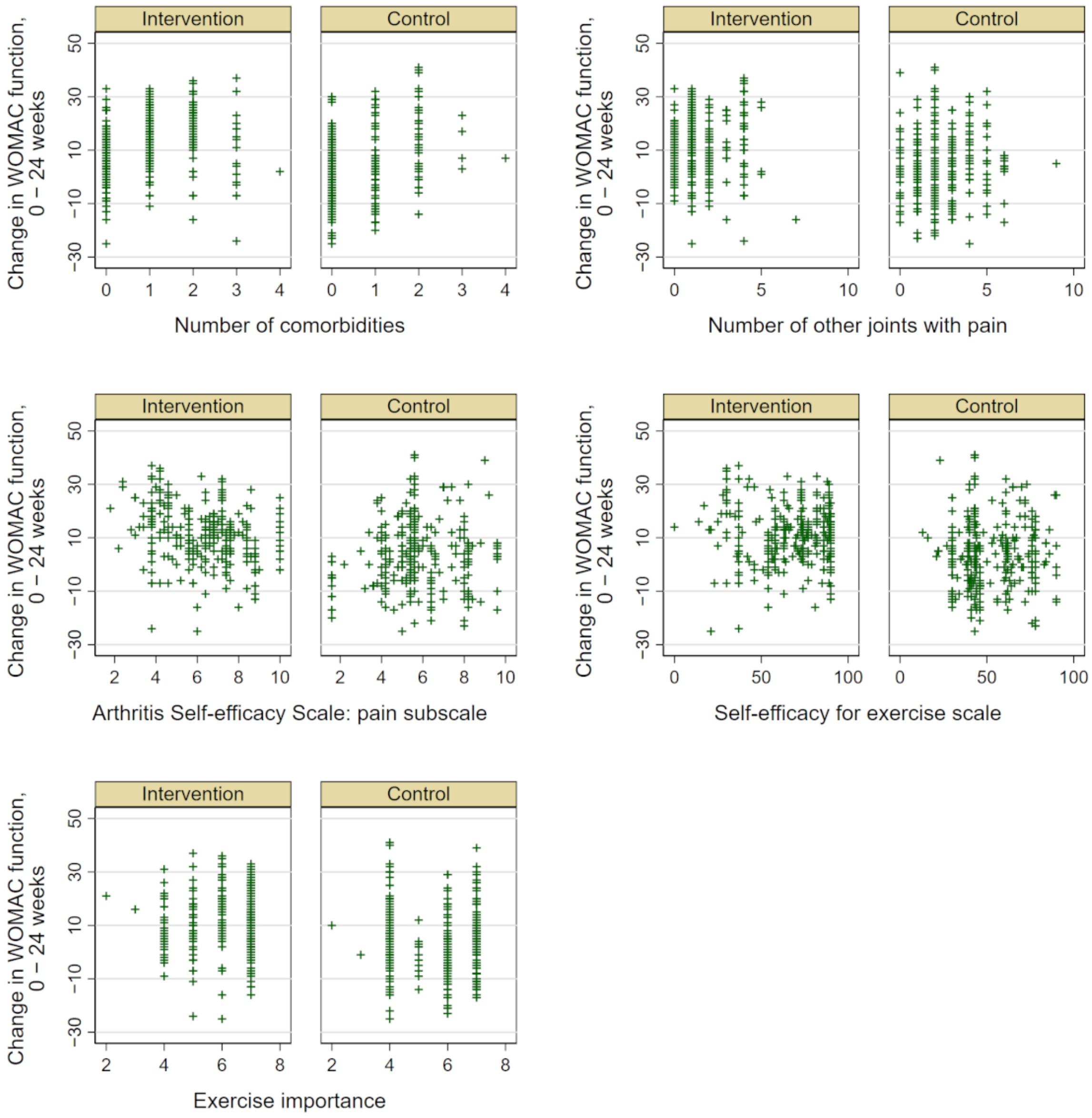

Supplement: Multimedia Appendix 2 [file jmir_v23i10e30768_app2.pdf]
