# Supplementary material for: Moderators of the Effect of a Self-directed Digitally Delivered Exercise Program for People With Knee Osteoarthritis: Exploratory Analysis of a Randomized Controlled Trial
Source: J Med Internet Res. 2021 Oct 29;23(10):e30768. doi: 10.2196/30768 (PMC8590189; doi:10.2196/30768)
Supplement: Multimedia Appendix 3 [file jmir_v23i10e30768_app3.pdf]

Multimedia Appendix 3: Results of the moderation analysis, presented in terms of the effect on the primary outcomes of a 1–unit increase in the moderators in each of the control and intervention groups using complete case data.

Outcome (change from baseline)	Moderator (taken at baseline)	Intervention Moderator Coeff. (95%CI)	P value	Control Moderator Coeff. (95%CI)	P value	Difference in coefficients, Intervention minus Control (95% CI)	Interaction P value
NRS overall pain	Number of comorbidities [a]	0.26 (–0.20 to 0.72)	0.26	0.16 (–0.32 to 0.64)	0.51	0.10 (–0.56 to 0.77)	0.760
WOMAC function		0.44 (–1.88 to 2.77)	0.71	2.36 (–0.04 to 4.76)	0.054	–1.92 (–5.25 to 1.41)	0.260
NRS overall pain	Number of other joints with pain [b]	–0.01 (–0.31 to 0.29)	0.96	0.10 (–0.15 to 0.35)	0.44	–0.11 (–0.50 to 0.29)	0.590
WOMAC function		–0.74 (–2.24 to 0.77)	0.33	0.94 (–0.36 to 2.24)	0.15	–1.68 (–3.67 to 0.31)	0.097
NRS overall pain	Arthritis Self–efficacy pain subscale [c]	0.23 (–0.04 to 0.50)	0.092	0.13 (–0.11 to 0.38)	0.29	0.10 (–0.26 to 0.46)	0.590
WOMAC function		–0.61 (–2.00 to 0.77)	0.38	1.53 (0.22 to 2.84)	0.022	–2.15 (–4.03 to –0.27)	0.025
NRS overall pain	Self–efficacy for exercise scale [d]	–0.00 (–0.02 to 0.02)	0.94	0.03 (0.00 to 0.05)	0.031	–0.03 (–0.06 to 0.00)	0.089
WOMAC function		0.03 (–0.07 to 0.14)	0.55	0.09 (–0.03 to 0.21)	0.15	–0.06 (–0.22 to 0.10)	0.470
NRS overall pain	Exercise importance [e]	0.09 (–0.26 to 0.45)	0.61	0.55 (0.18 to 0.92)	0.004	–0.46 (–0.97 to 0.05)	0.080
WOMAC function		0.10 (–1.74 to 1.93)	0.92	1.25 (–0.67 to 3.17)	0.20	–1.15 (–3.82 to 1.51)	0.390

[a]Collected via a question asking participants to select from a list of 13 comorbidities any of which were relevant to them. A participant's selected comorbidities were then added to create a continuous score of the total number of comorbidities per participant; this resulted in a range of 0 to 4.

[b]Collected via a question asking participants to select from a list of 9 joints any of which they currently experience pain in. Responses were converted into a continuous score of number of other joints with pain per participant, ranging from 0 to 9.

[c]Scores range from 1 to 10, with higher scores indicating greater self–efficacy for pain.

[d]Scores range from 0 to 90, with higher scores indicating greater self–efficacy for exercise.

[e]Measured via the response to the question "How important is it to you to do regular exercise to manage your knee condition?" Scores range from 1 to 7; higher score indicates higher importance.

NRS = numerical rating scale; WOMAC = The Western Ontario and McMaster Universities Osteoarthritis Index
