# Supplementary material for: Moderators of the Effect of a Self-directed Digitally Delivered Exercise Program for People With Knee Osteoarthritis: Exploratory Analysis of a Randomized Controlled Trial
Source: J Med Internet Res. 2021 Oct 29;23(10):e30768. doi: 10.2196/30768 (PMC8590189; doi:10.2196/30768)

Multimedia Appendix 6: Difference in mean change in NRS overall knee pain (baseline minus 24 weeks) between treatment groups (intervention minus control) for each potential continuous moderator, using complete case data. Positive values favor the intervention. Solid line indicates the difference between the control and intervention arms. Dashed line indicates no difference between the control and intervention arms. Shaded areas indicate 95% confidence intervals.

NRS = numerical rating scale

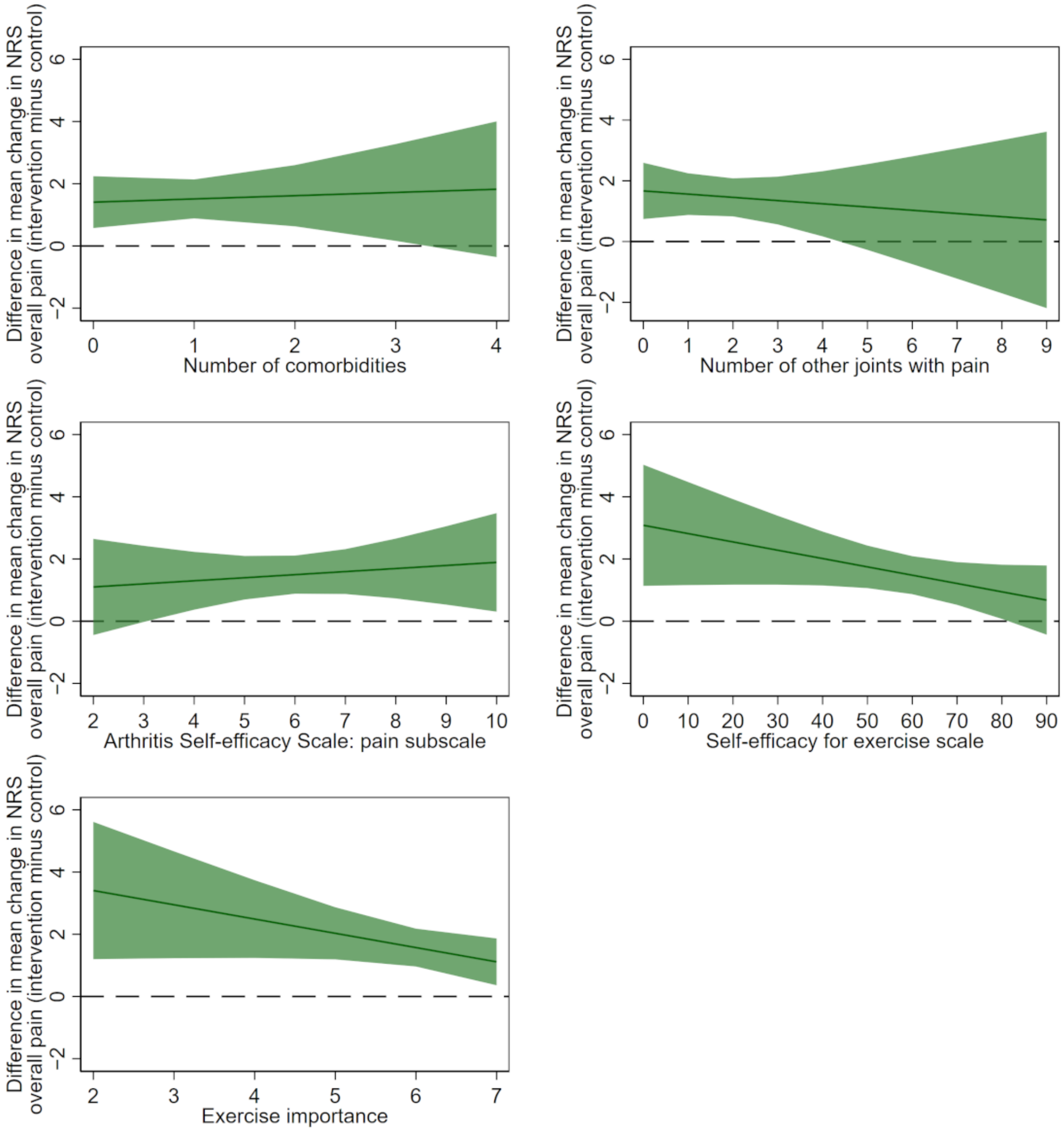

Supplement: Multimedia Appendix 6 [file jmir_v23i10e30768_app6.pdf]
